# Lipid nanoparticle-encapsulated DNA vaccine induces balanced antibody and T-cell responses in pigs with maternally derived antibodies

**DOI:** 10.1128/jvi.01123-25

**Published:** 2025-10-09

**Authors:** Danh C. Lai, The N. Nguyen, Giao P. Trinh, David Steffen, Hiep L. X. Vu

**Affiliations:** 1 Nebraska Center for Virology, University of Nebraska-Lincoln 14719, Lincoln, Nebraska, USA; 2 School of Veterinary Medicine and Biomedical Sciences, University of Nebraska-Lincoln 14719, Lincoln, Nebraska, USA; 3 Department of Animal Science, University of Nebraska-Lincoln 14719, Lincoln, Nebraska, USA; St Jude Children's Research Hospital, Memphis, Tennessee, USA

**Keywords:** LNP-DNA vaccine, MDA interference, swine influenza A virus, preweaning infection, young population

## Abstract

Maternally derived antibodies (MDAs) play a vital role in protecting neonates from infectious diseases, but their presence at the time of vaccination can interfere with vaccine-induced immune responses, thereby reducing vaccine effectiveness. MDA interference has been documented in pigs vaccinated with whole-inactivated virus (WIV) vaccines against swine influenza A virus (IAV). This study evaluated the efficacy of a lipid nanoparticle-encapsulated DNA (LNP-DNA) vaccine against swine IAV in the presence and absence of MDAs, comparing it to a WIV vaccine. In MDA-negative piglets, both the LNP-DNA and WIV vaccines induced strong immune responses and effectively prevented the vaccinated animals from being infected with the homologous IAV strain. However, in MDA-positive piglets, the WIV vaccine failed to trigger significant antibody or T-cell responses and offered no protection against viral shedding or lung damage. In contrast, the LNP-DNA vaccine elicited stronger immune responses in MDA-positive pigs, reduced nasal viral shedding, and prevented lung lesions. These findings demonstrate that the LNP-DNA vaccine overcomes MDA interference, making it a promising strategy for enhancing vaccine efficacy in neonatal animals with maternal antibodies.

**IMPORTANCE:**

Maternally derived antibody (MDA) interference is a major obstacle to developing effective vaccines for neonates. In pigs, MDAs significantly impair immune responses to a whole-inactivated virus vaccine. Here, we show that vaccination with a lipid nanoparticle (LNP)-encapsulated DNA vaccine can partially overcome MDA interference. These findings underscore the potential of the LNP-DNA vaccine as a viable strategy for effectively immunizing MDA-positive populations. Additionally, LNP-DNA vaccination in young pigs provides a valuable model for exploring the immunological mechanisms behind MDA-mediated suppression of vaccine-induced immunity.

## INTRODUCTION

Neonatal infections pose a major health risk in both humans and animals, as newborns are highly susceptible to infection (1). Maternally derived antibodies (MDAs), which are transferred to the offspring *in utero* and/or via breast milk, play a crucial role in protecting neonates during this critical period (2, 3). For certain high-risk pathogens, vaccinating during pregnancy is strongly recommended to enhance passive transfer of maternal immunity to the offspring (4). While MDAs provide essential early immunity, they may also hinder immune responses to vaccination (5). Major factors influencing the host immune response to immunization in the presence of MDAs include the nature and dose of vaccine immunogens, the routes of immunization, as well as the titers of MDAs at the time of vaccination (6). MDA interference has been frequently observed with the use of live-attenuated and viral-vector vaccines, as high MDA titers neutralize live agents in these vaccines, reducing or preventing their replication in the hosts and consequently impairing the induction of an active immune response (7–9). Similarly, MDA interference with non-replicating vaccines, including inactivated or protein-based vaccines, has been frequently documented (10–12). In many cases, MDAs appear to affect antibody responses more than T-cell responses (13, 14). The interference occurs through several mechanisms. MDAs may mask epitopes or cause steric hindrance, thus preventing their recognition by B cells (15). Additionally, MDAs may bind to vaccine antigens and promote their clearance via Fc-dependent phagocytosis. Alternatively, complexes of MDAs and vaccine antigens may cross-link the B-cell receptor with the FcγRIIB receptor, thereby inhibiting B-cell activation (16). In some instances, high titers of MDAs do not inhibit the formation of germinal centers in the draining lymph nodes or the induction of germinal center B cells (17). Instead, they inhibit the differentiation of B cells into plasma cells and/or memory B cells.

Although the results vary across experimental models, several studies have shown that immunization with DNA-based vaccines is less susceptible to interference by MDAs than other forms of vaccines (18, 19). This may be because the immunogens are delivered to the host as DNA molecules, which are not recognized by MDAs until protein synthesis occurs. Additionally, the vaccine immunogens are produced within host cells, where they are processed and presented to antigen-presenting cells differently from protein-based vaccines, which are extracellular antigens (20). Some studies also suggest that DNA vaccine immunogens can be expressed within the host for an extended period (21, 22), thereby continuously stimulating the host immune system, especially after the MDAs have waned (23).

Swine influenza A virus (IAV) is a major respiratory pathogen that significantly impacts swine production worldwide (24). IAV is classified based on two major envelope proteins: hemagglutinin (HA) and neuraminidase (NA). Currently, three IAV subtypes (H1N1, H1N2, and H3N2) are co-circulating in swine herds in the United States (25, 26). The substantial genetic and antigenic diversity within each subtype poses a significant challenge to the development of an efficacious vaccine (27). Furthermore, IAVs of human and avian origin frequently spill over into swine herds, and reassortment of genomic RNA segments between human, avian, and endemic swine IAVs can result in the emergence of novel strains, further complicating vaccine development efforts (28–33).

Currently, polyvalent whole-inactivated virus (WIV) vaccines are commonly used to control IAV in swine (34). While WIV vaccines provide protection against antigenically matched IAV strains, their effectiveness is significantly reduced when vaccinated animals are exposed to antigenically mismatched strains (35). Another limitation is that WIV vaccines fail to induce active immune responses when administered to piglets with MDAs (36, 37). Moreover, MDA-positive piglets vaccinated with a WIV vaccine and subsequently exposed to a heterologous IAV strain develop more severe lung lesions and clinical disease than non-vaccinated (NV) pigs, a phenomenon known as vaccine-associated enhanced respiratory disease (VAERD) (36, 38). Live-attenuated virus (LAV) vaccines had been licensed for clinical application in the United States. Under experimental conditions, LAV vaccines, when administered via the intranasal route, provide better heterologous protection than WIV vaccines (36, 39, 40). Unlike WIV vaccines, LAV vaccines partially protect MDA-positive pigs against challenges with heterologous IAV strains, rather than causing VAERD (36, 37). However, LAV vaccines carry the potential risk of reversion to virulence through reassortment with field IAV strains (41).

We recently developed and evaluated a novel lipid nanoparticle (LNP) formulation that efficiently encapsulates a DNA plasmid encoding the HA gene of H1N1 and H3N2 swine IAV (42, 43). In seronegative piglets, a single intramuscular immunization with the LNP-DNA vaccine elicited robust humoral and cellular immune responses and protected pigs from experimental challenges with the respective homologous IAV strains. In this study, we aimed to assess the immunogenicity and protective efficacy of the LNP-DNA vaccine against H1N2 in pigs with high titers of MDAs.

## MATERIALS AND METHODS

### Swine IAV

Swine influenza A/swine/Minnesota/A01392045/2013 (H1N2) (GenBank accession number: KF715130.1), belonging to the H1 1B.2.2.1 clade, was obtained from the National Veterinary Services Laboratories (NVSL, Ames, IA, USA). The virus was propagated in Madin-Darby canine kidney cells (MDCK-ATCC CCL-34) in infection medium (Dulbecco’s modified Eagle medium [DMEM, Gibco, #12800-058] supplemented with 0.2% bovine serum albumin fraction V [Sigma, #A8412], 25 mM HEPES [Hyclone, #SH30237.01], 100 U/mL penicillin and 100 µg/mL streptomycin [Gibco, #15140-122], and 1 µg/mL TPCK-treated trypsin [Sigma, #T1426]). After 60 h of incubation, the culture supernatant was collected, clarified by centrifugation at 3,000 × *g* for 10 min, aliquoted, and stored at −80°C. Virus titer was determined by titration on MDCK cells and expressed as log_10_ tissue infectious dose 50 (TCID_50_) per mL.

### Preparation of the LNP-DNA vaccine

The HA sequence of the swine IAV H1N2 (GenBank protein_id: AGZ62252.1) was codon-optimized for *Sus scrofa*. To facilitate protein detection, a FLAG-tag sequence (DYKDDDDK) was fused in-frame to the 3′ end of the gene. The gene fragment was synthesized using a commercial DNA synthesis service (GenScript) and cloned into the pCI plasmid backbone (Promega, #E1731) between the *NheI* and *NotI* sites. The recombinant plasmid was amplified in *E. coli* DH5α and purified using the Giga Prep Kit (Zymo Research, #D4204).

The LNP-DNA vaccine containing the HA gene was prepared as previously described (42, 43). The lipids used included DLin-MC3-DMA (MC3, Nanosoft Polymer, #1224606-06-7), 1,2-dioleoyl-3-trimethylammonium-propane (DOTAP, Avanti Polar Lipids, #132172-61-3), distearoylphosphatidylcholine (DSPC, Avanti Polar Lipids, #816-94-4), cholesterol (Sigma Aldrich, #57885), and 1,2-dimyristoyl-rac-glycero-3-methoxypolyethylene glycol-2000 (DMG-PEG2000, Avanti Polar Lipids, #160743-62-4). Each lipid was individually dissolved in absolute ethanol. Subsequently, MC3, DOTAP, DSPC, cholesterol, and DMG-PEG2000 were combined at a molar ratio of 42:10:8:38:2, respectively, to prepare the lipid mixture with a total lipid concentration of 15 mM. The DNA plasmid was diluted in 25 mM sodium acetate pH 4.0. The lipid and DNA solutions were combined using the Mixer-4 chip (PreciGenome, #CHP-MIX-4) under the control of a microfluidic device (NanoGenerator Flex-M, PreciGenome) at a DNA-to-lipid flow rate ratio of 3:1, with a total flow rate of 4 mL per minute. The nitrogen-to-phosphate ratio (mol/mol) was 5.5. The resulting products were dialyzed against Tris-Cl buffer pH 7.4 using Slide-A-Lyzer G2 dialysis cassettes with a 10 kDa molecular weight cutoff (Thermo Fisher Scientific, #87732) for 4 h at room temperature and overnight at 4°C. After dialysis, the LNPs were filtered through a 0.45 µm polyethersulfone membrane filter (Thermo Scientific, #09-740-114). The concentration of encapsulated DNA plasmid was quantified using a Quant-iT PicoGreen dsDNA Assay Kit (Invitrogen, #P7589), following the procedure described previously (44). Each dose of the LNP-DNA vaccine contained 500 µg of encapsulated DNA plasmid in a 5 mL volume. The vaccine was administered intramuscularly to pigs on the same day of preparation.

### Preparation of the WIV vaccine

To inactivate the virus, β-propiolactone (BPL, Sigma-Aldrich, #P5648) was added to the virus stock at a final concentration of 0.05% (vol/vol), followed by continuous stirring overnight at 4°C. The next morning, the BPL was hydrolyzed by incubation at 37°C for 2 h. To confirm complete inactivation, the virus was inoculated onto MDCK cells for three successive rounds. The inactivated virus stock was purified by ultracentrifugation on a 20% sucrose cushion in the NTC buffer (100 mM NaCl, 20 mM Tris/Cl pH 7.4, and 5 mM CaCl_2_) at 175,000 × *g*, using a SW32Ti rotor (Beckman Coulter) for 2 h at 4°C. The resulting virus pellet was resuspended in sterile phosphate-buffered saline (PBS, pH 7.4). Virus titers were determined using a hemagglutination (HA) assay. The virus concentration was adjusted in PBS and mixed with the Emulsigen-DL 90 adjuvant (Phibro Animal Health) at a 4:1 (vol/vol) ratio. The vaccine dose for sows contained 512 HA units in a 2 mL volume, while the vaccine for piglets contained 32 HA units, also in a 2 mL volume. In both cases, the vaccine was administered intramuscularly to pigs on the same day of preparation.

### Animal experiment

Four sows were procured from the University of Nebraska-Lincoln (UNL) Swine Research Farm and housed in the animal biosafety level 2 (ABSL-2) research facility at UNL. These sows were tested negative for antibodies against porcine reproductive and respiratory syndrome virus (PRRSV) and swine IAV using commercial ELISA kits purchased from IDEXX Laboratories (PRRS X3, #99-18070 for PRRSV; and Swine Influenza AB, #99-0000900 for IAV). After 1-week acclimation, two sows were vaccinated intramuscularly with 2 doses of the WIV vaccine 44 and 23 days before the expected parturition. The remaining two sows received no treatment and served as NV controls. Serum samples were collected from the sows every 2 weeks following vaccination to monitor antibody responses. All sows farrowed naturally, and the piglets were allowed to nurse from their own dams.

On day 14 post-farrowing, blood samples were drawn from all piglets and serum was collected to evaluate for the hemagglutination inhibition (HI) antibody titers. On day 21 post-farrowing, the piglets were administered a dose of ceftiofur crystalline-free acid to mitigate the risk of respiratory bacterial infections and were subsequently weaned. During the weaning, 18 piglets nursed by the two WIV-vaccinated sows, which exhibited HI titers between 1:320 and 1:640 at 14 days of age, were selected and assigned to three groups: MDA^(+)^/LNP, MDA^(+)^/WIV, and MDA^(+)^/NV (Table 1). Similarly, 18 piglets nursed by the NV sows were selected and allocated into three groups: MDA^(−)^/LNP, MDA^(−)^/WIV, and MDA^(−)^/NV. Each treatment group within the MDA-positive and MDA-negative categories received the same number of piglets from each respective sow.

**TABLE 1:** Animal study design

Treatment group	MDA status^*a*^	Vaccine	Prime	Boost	Number of pigs
MDA^(−)^/NV	–	None	N/A^*b*^	N/A	6
MDA^(−)^/LNP	–	LNP-DNA	500 µg	N/A	6
MDA^(−)^/WIV	–	WIV	32 HAU	32 HAU	6
MDA^(+)^/NV	+	None	N/A	N/A	6
MDA^(+)^/LNP	+	LNP-DNA	500 µg	N/A	6
MDA^(+)^/WIV	+	WIV	32 HAU	32 HAU	6

^*a*^ +, MDA positive; –, MDA negative.

^*b*^ N/A, not applicable.

On day 28 post-farrowing, day 0 post-vaccination, the MDA^(+)^/LNP and MDA^(−)^/LNP groups received an intramuscular injection of the LNP-encapsulated DNA plasmid. Meanwhile, the MDA^(+)^/WIV and MDA^(−)^/WIV groups received two intramuscular injections of the WIV vaccine, administered 21 days apart (days 0 and 21 post-vaccination). The remaining groups, MDA^(+)^/NV and MDA^(−)^/NV, received no treatment and served as NV controls (Table 1).

On day 36 post-vaccination, the pigs were challenged with 2 × 10^5^ TCID_50_ of the homologous H1N2 virus, via both intratracheal and intranasal routes, as previously described (43). On day 5 post-challenge, the pigs were humanely euthanized with an overdose of sodium pentobarbital (Fatal-Plus Solution, Vortech Pharmaceutical). Necropsies were performed by a board-certified pathologist, who is one of the co-authors and was intentionally blinded to the animal treatment groups.

### Sample collection

Whole blood samples were collected with and without an anticoagulant before vaccination and on days 14, 21, 28, and 35 post-vaccination and day 5 post-challenge, corresponding to day 41 post-vaccination. Serum samples were isolated from blood collected without an anticoagulant to measure antibody responses. Peripheral blood mononuclear cells (PBMCs) were isolated from blood samples collected in EDTA tubes by density gradient centrifugation using Lymphoprep density gradient medium (STEMCELL Technologies, #07851) and SepMate−50 tubes (STEMCELL Technologies, #85450). After isolation, cell density was counted, and the cells were cryopreserved in a freezing medium consisting of 40% RPMI-1640 (Gibco, # 61870036), 50% fetal bovine serum (FBS; Sigma-Aldrich, #12,107C), and 10% dimethyl sulfoxide (DMSO; Sigma-Aldrich, #D2650).

Nasal swabs were collected daily post-challenge, using sterile ultrafine polyester flocked swabs (Puritan, #25-3317-U) and immersed in 1 mL of DMEM containing 100 units/mL penicillin and 100 µg/mL streptomycin. The samples were centrifuged at 3,000 × *g* for 10 min to remove debris, aliquoted into two tubes of approximately 400 µL each, and stored at –80°C until use.

Oral swabs were collected prior to challenge (i.e., day 36 post-vaccination). Additionally, tracheal swabs were collected during necropsy. Sterile 4 × 4 cotton gauze pads (Gauze Care) were used for sample collection. Each pad was placed in a 50 mL conical tube containing 3 mL of cold PBS. Subsequently, the pad was transferred into a 30 mL syringe (Exelint International, #26290), and the plunger was used to compress the pad. The recovered fluid was collected and centrifuged at 3,000 × *g* for 10 min to remove debris. The clarified supernatant was aliquoted into small volumes and stored at −80°C until use.

Bronchoalveolar lavage fluid (BALF) was collected during necropsy. After the lungs were removed from the chest cavity, the trachea was cut away near the bifurcation. Fifty milliliters of cold PBS was instilled into the lung, followed by gentle massage to distribute the fluid. The lavage fluid was then recovered, centrifuged at 3,000 × *g* for 10 min, and stored at –80°C until use.

A Bio-Thermo microchip (Merck Animal Health, #362118) was implanted intramuscularly in the neck muscle of the pigs to facilitate body temperature measurement. Body temperature was recorded daily using a Global pocket Reader Plus (Destron Fearing, model GPR+) from 1 day before challenge to day 5 post-challenge.

### Pathological analysis and *in situ* hybridization (ISH) assay

Gross lung lesions were scored during necropsy. The percentage of lung surface exhibiting consolidation typical of IAV infection was estimated for each lung lobe. The total lung consolidation was then calculated based on the weighted proportions of each lobe relative to the total lung volume (45, 46).

During necropsy, samples from the trachea and the apical, middle, and caudal lung lobes were collected and fixed in 10% buffered formalin for 24 h. The samples were then submitted to the Nebraska Veterinary Diagnostic Center, where they were embedded in paraffin and sectioned at a thickness of 4 µm. Lung sections were stained with hematoxylin and eosin (H&E) for histological evaluation. Lung microscopic lesions were scored based on four parameters proposed by Gauger et al. (47), including necrotizing, suppurative bronchitis and bronchiolitis, peribronchiolar lymphocytic cuffing, and alveolar septal inflammation. An additional parameter, microscopic lung consolidation, was introduced in this study. Each parameter was scored according to the percentage of cross-sectional area affected, using a scale of 0–3, where 0 = normal, 1 = <5%, 1.5 = 5–25%, 2.0 = 26–50%, 2.5 = 51–75%, and 3.0 = >75% affected. The total score (maximum of 15) was calculated for each lung lobe, and the average across the three lobes was reported per animal.

Additionally, sections of the cardiac lung lobe and trachea were subjected to RNA ISH using a probe specific to viral nucleoprotein (NP) transcripts (Advanced Cell Diagnostics, #579001) and the RNAscope 2.5 HD Reagent Kit-BROWN (Advanced Cell Diagnostics, #322300), as previously described (48). The ISH score was assigned based on the abundance of virus-infected cells within a microscopic field at 40× objective magnification, as previously described (49). Scale: 0 = none; 1 = 1–10 cells; 2 = 11–30 cells; 3 = 31–100 cells; and 4 = >100 cells.

### HI assay

Serum samples were first incubated at 56°C for 30 min. The samples were then mixed at a 1:4 (vol/vol) ratio with 20% kaolin and incubated for 20 min at room temperature, followed by centrifugation at 800 × *g* for 5 min. Subsequently, the supernatants were incubated with an equal volume of 0.5% turkey red blood cells (RBCs, Lampire Biological Laboratories, #7209403) for 20 min at room temperature, followed by centrifugation at 400 × *g* for 5 min to remove non-specific inhibitors. The treated sera were then serially diluted twofold in PBS in a 96-well V-bottom plate (Greiner Bio-One, #651101), starting at a 1:10 dilution. An equal volume of the H1N2 virus containing 4 HA units was added and incubated for 30 min. Subsequently, 0.5% turkey RBCs were added, and the plates were incubated for 1 hour at room temperature. HI titers were determined as the reciprocal of the highest serum dilution, which completely inhibited hemagglutination.

### Influenza NP antibody assay

Antibodies specific to the influenza NP were detected using a blocking ELISA kit (IDEXX Laboratories, Swine Influenza AB, #99-0000900), following the manufacturer’s instructions. As a blocking enzyme-linked immunosorbent assay (ELISA), color development is inversely proportional to concentrations of NP-specific antibodies in the test samples. Results were expressed as the ratio of the optical density (OD) of the test sample to the negative control (*S*/*N* ratio). Samples with an *S*/*N* ratio below 0.6 were considered positive.

### HA-specific indirect ELISAs

An indirect ELISA was developed to assess HA-specific IgG and IgA antibodies in oral swabs. To produce the HA antigen for coating the ELISA plates, HEK-293T cells cultured in a 100 mm tissue culture dish (TPP Techno Plastic Products, #93100) were transfected with 30 µg of the plasmid containing the HA gene of the swine IAV H1N2 virus, the same construct used to formulate the LNP vaccine. At 48 h post-transfection, the cells were harvested and lysed in 1,200 µL of Pierce RIPA buffer (Thermo Fisher Scientific, #89901). The resulting lysate was dialyzed overnight at 4°C against PBS using a Slide-A-Lyzer G2 dialysis cassette, MWCO of 10 kDa (Thermo Fisher Scientific, #87730). The total protein concentration of the lysate was quantified using the bicinchoninic acid protein assay kit (Thermo Fisher Scientific, #23227).

To coat the ELISA plates, the protein lysate was diluted in 0.1 M carbonate/bicarbonate buffer (pH 9.6) to a final concentration of 6.25 µg/mL. A volume of 100 µL of the diluted protein was added to each well of LockWell Maxisorp ELISA plates (Thermo Fisher Scientific, #446469) and incubated overnight at 4°C. The plates were washed with tris-buffered saline, pH 7.4, containing 0.1% Tween 20 (TBS-T20), followed by blocking at room temperature for 1 h using a sample dilution buffer (ChonBlock blocking/sample buffer [Chondrex Inc, #9068] diluted 1:1 with TBS-T20). Oral swab samples were diluted 1:4 in the sample dilution buffer. Then, 100 µL of the diluted samples was added in duplicate to each well of the ELISA plates and incubated for 1 h at room temperature. After five washes with TBS-T20, goat anti-pig IgG (H + L) HRP-conjugated (Bethyl Laboratories, #A100-105P) or goat anti-pig IgA HRP-conjugated (Bio-Rad, #AAI40P) secondary antibodies were added and incubated for 1 h. Both antibodies were diluted at 1:2,000 in the sample dilution buffer. Following five more washes with TBS-T20, the reaction was developed with TMB (3,3′,5,5′-tetramethylbenzidine) substrate (Alpha Diagnostic International, #80093-1000) for 7 min, stopped with a stop solution (Alpha Diagnostic International, #80100), and absorbance was measured at 450 nm using Biotek Synergy LX Plate Reader (BioTek Instruments Inc.).

### Interferon-gamma (IFN-γ) ELISpot assay

The IFN-γ ELISpot assay was performed, as previously described (48). Sterile 96-well filter plates with 0.45 µm pore size hydrophobic polyvinylidene fluoride membranes (Millipore Sigma, #MSIPS4W10) were coated with 50 µL/well of anti-porcine IFN-γ antibody (BD Biosciences Pharmingen, clone P2G10, #559961) at a concentration of 10 µg/mL overnight at 4°C. Plates were washed three times with sterile PBS and blocked by incubation with 250 µL/well of complete RPMI (RPMI-1640 supplemented with 10% FBS, 100 U/mL penicillin, and 100 µg/mL streptomycin) for 2 h at 37°C in a 5% CO_2_ incubator.

Cryopreserved PBMCs were thawed, washed, and resuspended in complete RPMI, and 100 µL containing 500,000 cells were added to each well. Six wells were used per PBMC sample. Two wells were stimulated with the H1N2 virus at a concentration of 10^6^ TCID_50_ per well in 100 µL of complete RPMI. Two wells were stimulated with a cocktail of phorbol 12-myristate 13-acetate (PMA; Sigma-Aldrich, #P8139, final concentration 10 ng/mL) and ionomycin (Sigma-Aldrich, #I0634, final concentration 1 µg/mL), diluted in 100 µL of complete RPMI, as the positive control. The remaining two wells received 100 µL of complete RPMI and served as unstimulated controls.

After 20 h of incubation at 37°C in a 5% CO_2_ incubator, the cells were removed, and plates were washed three times with 250 µL per well of PBS containing 0.05% Tween 20 (PBS-T20). Fifty microliters of biotinylated mouse anti-pig IFN-γ antibody (BD Biosciences Pharmingen, #559958), diluted to a final concentration of 2 µg/mL in PBS-T20, was added to each well, followed by incubation at room temperature for 1 h. After five washes with PBS-T20, plates were incubated with streptavidin-conjugated alkaline phosphatase (Southern Biotech, #7105-04), diluted 1:1,000 in PBS-T20. Following another five washes with PBS-T20, plates were incubated with alkaline phosphatase substrate (Vector Laboratories, #SK-5300) for 15 min. Color development was stopped by washing with water. Spots were analyzed and counted using the CTL Immunospot S5 Analyzer (ImmunoSpot). The number of spots in wells stimulated with H1N2 virus was subtracted from the number of spots in unstimulated wells. Data are presented as the number of IFN-γ-secreting cells (IFN-γ-SC) per 5 × 10^5^ PBMCs.

### Virus titration

Virus titers in the nasal swabs and BALF samples were determined by titration in MDCK cells, as previously described (50). The titers were calculated using the Reed and Muench method and expressed as the log_10_ TCID_50_ per mL. Samples with undetected infectious virus were assigned a value of 0 log_10_ for graphical presentation and statistical analysis.

### Statistics

Graphical and statistical analyses were conducted using GraphPad Prism 10 (GraphPad Software Inc.). Prior to statistical testing, HI titers and viral infectious titers were log-transformed using base 2 and base 10, respectively. Continuous single-variable data were analyzed using Brown-Forsythe and Welch’s ANOVA, followed by unpaired *t*-tests with Welch’s correction. For continuous two-variable data, including HI titers in piglets post-vaccination and viral titers following challenge, two-way ANOVA was performed, followed by Tukey’s multiple comparisons test. Ranked data, including H&E and ISH scores, were analyzed using the Kruskal-Wallis test, followed by uncorrected Dunn’s multiple comparisons test.

## RESULTS

### Generation of piglets with and without colostrum-transferred IAV-specific maternal antibodies

**Fig 1:**
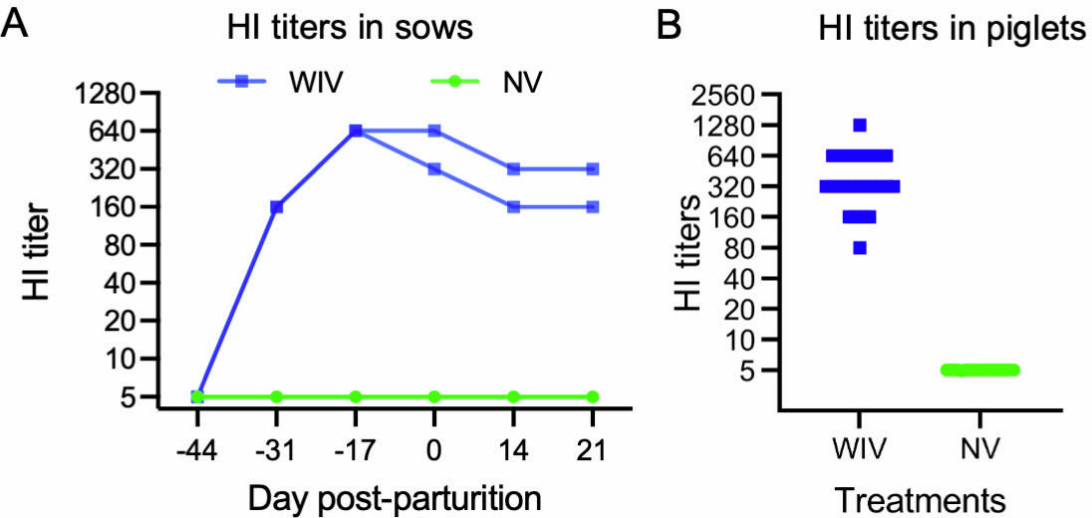
HI titers in sows and piglets. (**A**) Two sows were vaccinated with two doses of the WIV vaccine, while two other sows served as NV controls. Blood samples were collected at the indicated time points, and HI titers against the H1N2 virus were measured. (**B**) Blood samples were drawn on day 14 post-farrowing from piglets nursed by WIV-vaccinated and NV sows. HI titers against the H1N2 virus were measured. Symbols represent data from individual animals. Samples with undetectable HI antibodies were assigned a titer of 1:5 for graphical presentation.

To produce piglets with and without IAV-specific MDAs, two sows were administered two doses of the WIV vaccine on days 44 and 23 before the expected parturition, while two other sows served as NV controls. The WIV-vaccinated sows developed high HI antibody titers, reaching 1:640 on day 17 before parturition. On the day of weaning, 21 days post-parturition, the HI antibody titers of the two WIV-vaccinated were still relatively high, measuring at 1:160 and 1:320, respectively. The NV control sows had no detectable HI titers throughout the study (Fig. 1A). As a result, piglets nursed by the WIV-vaccinated sows had high HI titers in their serum on day 14 after birth, with the titers ranging from 1:80 to 1:1,280. Conversely, piglets nursed by the NV sows had no detectable HI titers (Fig. 1B).

### Antibody responses after vaccination

**Fig 2:**
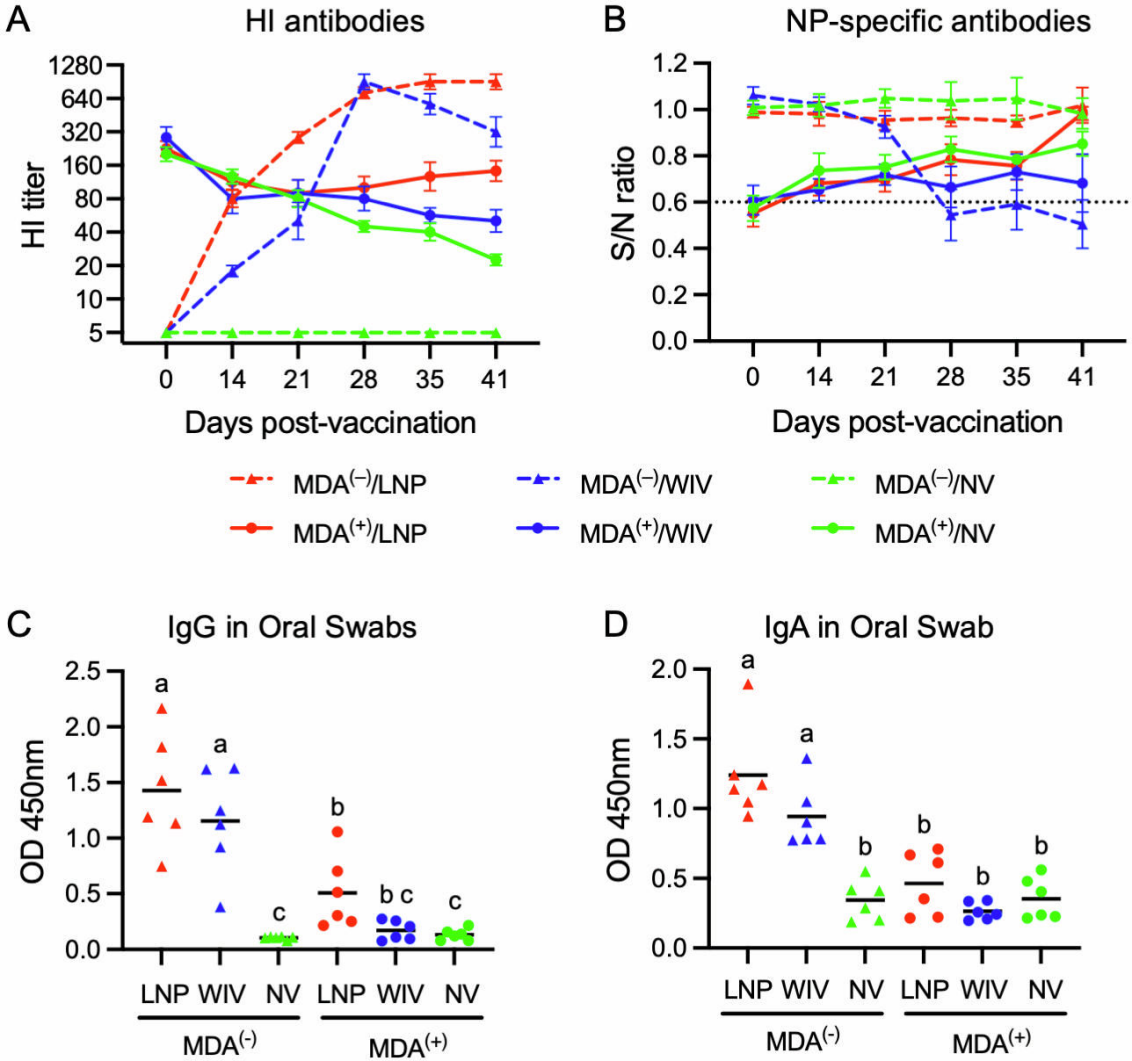
Antibody responses in piglets after vaccination. Piglets with and without MDAs were either kept as non-vaccinated (NV) controls or vaccinated with either the WIV or LNP-DNA vaccine at day 28 of age. Serum samples were collected at the indicated days post-vaccination to measure systemic antibody responses. Day 41 post-vaccination corresponds to day 5 post-challenge. (**A**) Serum HI titers against the H1N2 virus. Samples with undetectable HI antibodies were assigned a titer of 1:5 for graphical presentation. Data are presented as geometric mean titer ± standard error of the mean (SEM). Statistical analysis is presented in Table S1. (**B**) Antibodies specific to the viral nucleoprotein (NP) measured by a blocking ELISA. Data are presented as sample-to-negative (*S*/*N*) ratios, which are inversely correlated with antibody concentration in the test samples. The dotted line at *S*/*N* = 0.6 indicates the assay cutoff, with samples below this threshold considered positive. (**C and D**) HA-specific IgG and IgA antibodies in oral swabs collected on day 36 post-vaccination. HA-specific IgG and IgA were measured by indirect ELISA, and data are presented as optical density (OD) at 450 nm. Symbols represent data from individual animals while lines represent the group means. Treatment groups with different superscripts are significantly different (*P* < 0.05).

Eighteen piglets from NV- and 18 piglets from WIV-vaccinated sows were selected for a vaccination study. At the time of vaccination, 28 days of age, all 18 piglets from the NV sows had undetectable HI titers (Fig. 2A). Of these, the six piglets assigned to the MDA^(–)^/NV group maintained undetectable HI titers throughout the study. In the MDA^(–)^/LNP group, the mean HI titer was 1:80 on day 14 post-vaccination and continued to rise, reaching 1:640 by day 35 post-vaccination. In the MDA^(−)^/WIV group, the mean HI titer was 1:18 on day 14 post-vaccination, sharply increased to 1:960 on day 28, corresponding to 7 days after the second immunization, and then declined. The mean HI titers in the MDA^(−)^/LNP group were statistically higher than those in the MDA^(−)^/WIV group on days 14, 21, and 41 post-vaccination (Table S1).

At the time of vaccination (day 0), the 18 piglets selected from WIV-vaccinated sows had HI titers ranging from 1:160 to 1:640, with the mean HI titers not significantly different among the groups (Fig. 2A). The HI titers of the MDA^(+)^/NV and MDA^(+)^/WIV groups steadily declined at a similar rate, reaching mean titers of 1:22 and 1:50, respectively, on day 41 post-vaccination, corresponding to day 5 post-challenge. Interestingly, in the MDA^(+)^/LNP group, the HI titer initially dropped to a mean of 1:90 by day 21 post-vaccination but gradually increased afterward, reaching a mean titer of 1:143 by day 41 post-vaccination. On days 35 and 41 post-vaccination, the HI titers in the MDA^(+)^/LNP group were statistically higher than those in the MDA^(+)^/WIV group (Fig. 2A and Table S1).

Serum antibodies specific to the viral NP were measured using a commercially available ELISA kit, which is widely used for IAV serodiagnosis. On day 0 post-vaccination, NP-specific antibodies were not detected in any of the 18 pigs from the NV control sows (i.e., MDA-negative pigs) (Fig. 2B). Additionally, NP-specific antibodies were not detected at any time in the MDA^(−)^/LNP group throughout the study. In the MDA^(−)^/WIV group, NP-specific antibodies were detected on day 28 post-vaccination, 7 days after the second immunization. These results were expected as the NP antigen was included in the WIV but not the LNP-DNA vaccine. On day 0 post-vaccination, NP-specific antibodies were detected in 13 of 18 pigs born to WIV-vaccinated sows, although their S/N values were near the detection threshold (Fig. 2B). In the MDA^(+)^/NV and MDA^(+)^/LNP groups, the maternally derived NP-specific antibodies gradually declined over time and became undetectable by the NP-based ELISA by day 14 post-vaccination. Similarly, the NP-specific antibodies in the MDA^(+)^/WIV group also gradually declined and became undetectable, even though this group received two doses of the WIV which contained the NP antigen, suggesting that MDAs hindered the development of NP-specific antibody responses in these piglets (Fig. 2B).

To access the presence of antigen-specific antibodies at the mucosal surface, oral swabs were collected on day 36 post-vaccination, and HA-specific IgG and IgA antibodies were measured using indirect ELISAs. In MDA-negative pigs, the OD values for IgG and IgA in both the MDA^(−)^/LNP and MDA^(−)^/WIV groups were significantly higher than those in the MDA^(−)^/NV group (Fig. 2C and D). However, in MDA-positive pigs, only the MDA^(+)^/LNP group showed significantly higher IgG OD values compared to the MDA^(+)^/NV group. The OD values for IgA, however, were not significantly different among the MDA^(+)^ groups.

### T-cell responses after vaccination

**Fig 3:**
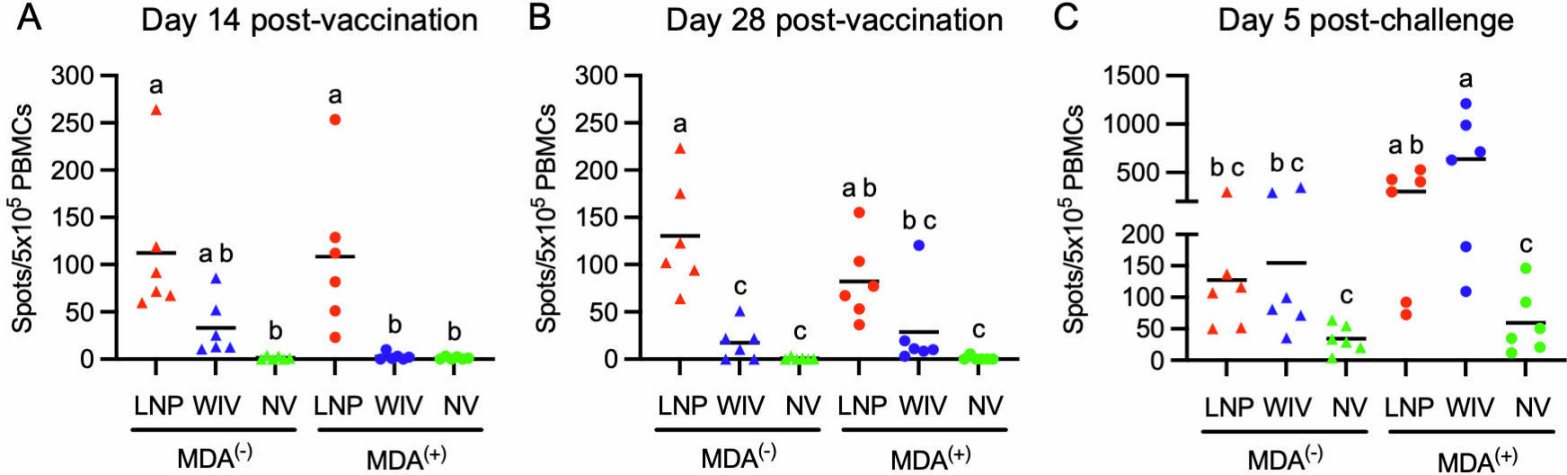
T-cell responses in piglets. PBMCs collected on days 14 (**A**) and 28 (**B**) post-vaccination and day 5 post-challenge (**C**) were stimulated with the H1N2 virus. The number (IFN-γ-SC) was quantified using the IFN-γ ELISpot assay. Data are presented as the number of spots per 5 × 10^5^ cells after background subtraction. Samples with negative spot counts following background subtraction were assigned a value of 0 for graphical presentation and statistical analysis. Symbols represent data from individual animals and lines represent group means. Treatment groups with different superscripts are significantly different (*P* < 0.05).

The IFN-γ ELISpot assay was used to evaluate T-cell responses. IFN-γ-SC were virtually undetectable in PBMCs collected from the MDA^(+)^/NV and MDA^(−)^/NV groups on days 14 and 28 post-vaccination (Fig. 3; Fig. S1), indicating the absence of maternally transferred virus-specific T cells. The WIV vaccine induced low but detectable T-cell responses in MDA-negative pigs. In the MDA^(−)^/WIV group, the mean numbers of IFN-γ-SC were 33 and 17 per 5 × 10⁵ PBMCs on days 14 and 28 post-vaccination, respectively. However, due to substantial inter-individual variability, these responses were not statistically different from those observed in the NV groups. In the MDA^(+)^/WIV group, the corresponding values were 3 and 29 cells per 5 × 10⁵ PBMCs. In contrast, the LNP-DNA vaccine induced stronger T-cell responses in both MDA-negative and MDA-positive pigs. On both days 14 and 28 post-vaccination, the mean numbers of IFN-γ-SC in the MDA^(−)^/LNP and MDA^(+)^/LNP groups were statistically higher than those in the NV groups. The means IFN-γ-SC in the MDA^(+)^/LNP were slightly lower than those in the MDA^(−)^/LNP group (108 vs 112 on day 14 and 82 vs 130 on day 28), but these differences were not statistically significant. Following challenge infection with the H1N2 virus, the numbers of IFN-γ-SC increased across all treatment groups, with the most pronounced increase observed in the MDA^(+)^/LNP and MDA^(+)^/WIV groups, indicating an anamnestic T-cell response.

Collectively, these results demonstrate that a single dose of the LNP-DNA vaccine induces high T-cell responses regardless of MDA status, whereas the WIV vaccine elicits weaker T-cell responses, even in MDA-negative pigs. However, the WIV vaccine effectively primes the immune system, leading to a strong anamnestic T-cell response following challenge.

### Body temperature after challenge

**Fig 4:**
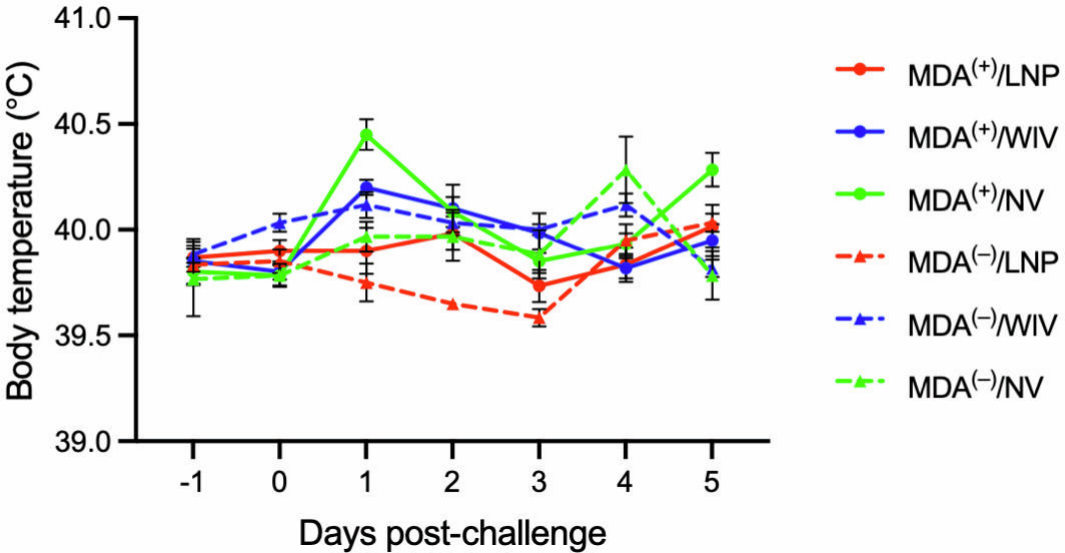
Body temperature. A Bio-Thermo microchip was injected intramuscularly into the neck muscle of the pigs. Body temperature was recorded daily from 1 day before challenge to day 5 post-challenge using a scanner. Data are presented as mean ± standard error of the mean.

The IAV strain used for the challenge did not induce a significant febrile response. The body temperature after infection ranged between 39°C and 40°C (Fig. 4), remaining below the 40.5°C threshold, which is considered indicative of fever.

### Viral loads after challenge infection

**Fig 5:**
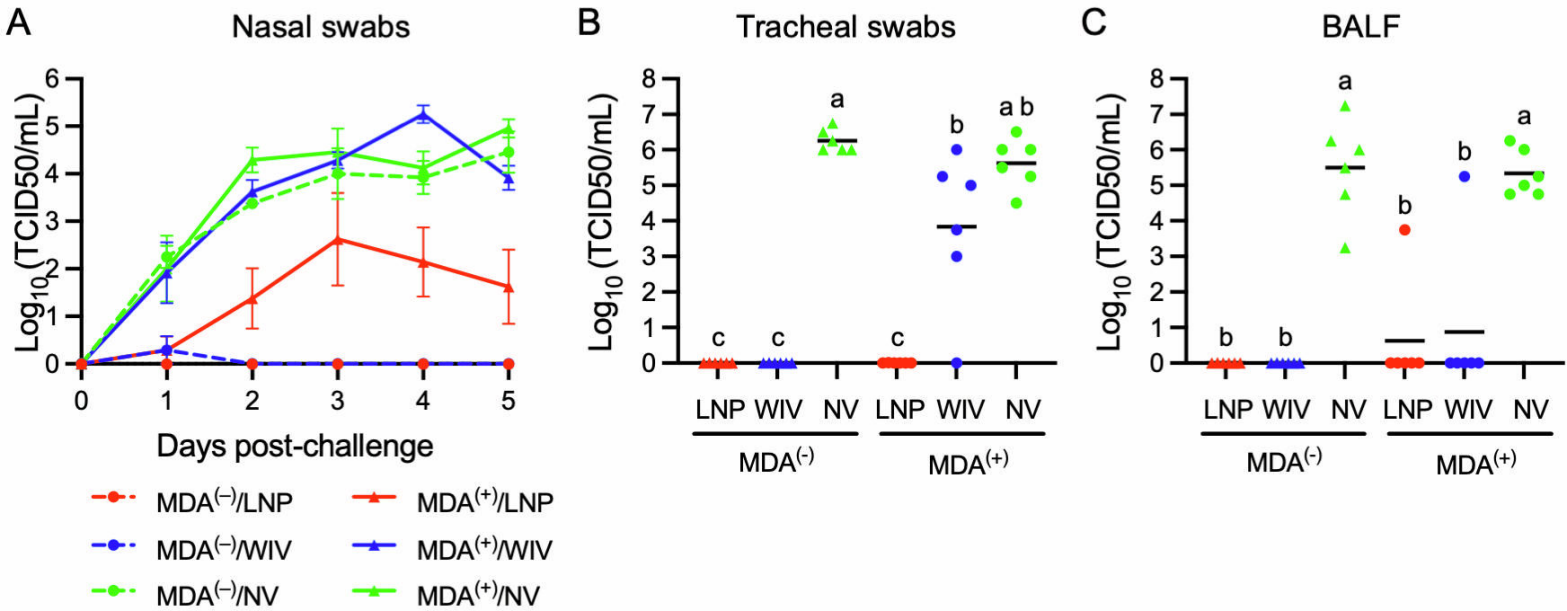
Infectious virus titers. Nasal swabs were collected daily post-challenge with the H1N2 virus, while tracheal swabs and BALF samples were collected during necropsy on day 5 post-challenge. Infectious virus in the samples was titrated in MDCK cells. (**A**) Infectious virus titers in nasal swabs. Data are presented as mean ± standard error of the mean. Statistical analysis is presented in Table S2. (**B and C**) Virus titers in tracheal swabs and BALF samples. Symbols represent data from individual samples, and the line represents the means. Treatment groups with different superscripts are significantly different (*P* < 0.05).

Nasal swabs were collected from all pigs following challenge inoculation with the H1N2 virus to assess viral shedding. In both MDA^(+)^/NV and MDA^(−)^/NV groups, infectious virus was detected in nasal swabs on day 1 post-challenge, with mean titers of 10^2^ and 10^2.2^ TCID_50_/mL, respectively (Fig. 5A). Virus titers increased through day 5 post-challenge, reaching mean titers of 10^5^ and 10^4.5^ TCID_50_/mL, with no statistically significant difference observed between the two groups. In MDA-negative pigs, both the LNP-DNA and WIV vaccines effectively prevented viral shedding. Infectious virus was detected in only one swab from the MDA^(−)^/WIV group on day 1 post-challenge, while infectious virus was not detected in nasal swabs from the MDA^(−)^/LNP group throughout the 5-day observation period. In MDA-positive pigs, the WIV vaccine failed to prevent viral shedding in nasal secretions. Infectious virus was consistently detected in nasal swabs from the MDA^(+)^/WIV group throughout the 5-day observation period, with shedding kinetics and magnitudes similar to those of the NV control groups. In the MDA^(+)^/LNP group, infectious virus was also detected in nasal swabs, with mean titers ranging from 10^0.3^ TCID_50_/mL on day 1 to 10^3.9^ TCID_50_/mL on day 5 post-challenge. However, these titers were significantly lower than those in both the NV control groups and the MDA^(+)^/WIV group (Fig. 5A and Table S2).

During necropsy, tracheal swabs were collected to evaluate the presence of infectious virus in the lower respiratory tract. Virus titers ranging from 10^4^ to 10^7^ TCID_50_/mL were detected in the swabs from both NV control groups, with no statistically significant difference between them (Fig. 5B). Infectious virus was not detected in any swabs from the MDA^(−)^/WIV, MDA^(−)^/LNP, and MDA^(+)^/LNP groups. In the MDA^(+)^/WIV group, five of six swabs contained infectious virus, with titers ranging from 10^3^ to 10^6^ TCID_50_/mL. The mean virus titers in this group were significantly lower than those in the MDA^(−)^/NV control group.

During necropsy, BALF samples were also collected to quantify the presence of the virus in the bronchoalveolar space. Infectious virus was detected in the BALF from both MDA^(−)^/NV and MDA^(+)^/NV groups, with a mean titer of 10^5^.^75^ and 10^5.13^ TCID_50_/mL, respectively (Fig. 5C). In contrast, no infectious virus was detected in any samples from the MDA^(−)^/LNP and MDA^(+)^/LNP groups. Infectious virus was found in only one sample from each of the MDA^(+)^/LNP and MDA^(+)^/WIV groups.

Overall, in MDA-negative pigs, both the LNP-DNA and WIV vaccines effectively prevented infection. In MDA-positive pigs, the WIV vaccine failed to prevent viral shedding in nasal secretions and the tracheal tract but successfully protected the lungs from infection. In contrast, the LNP-DNA vaccine significantly reduced viral shedding in nasal secretions and nearly prevented viral replication in the tracheal tract and lungs.

### Lung pathology

**Fig 6:**
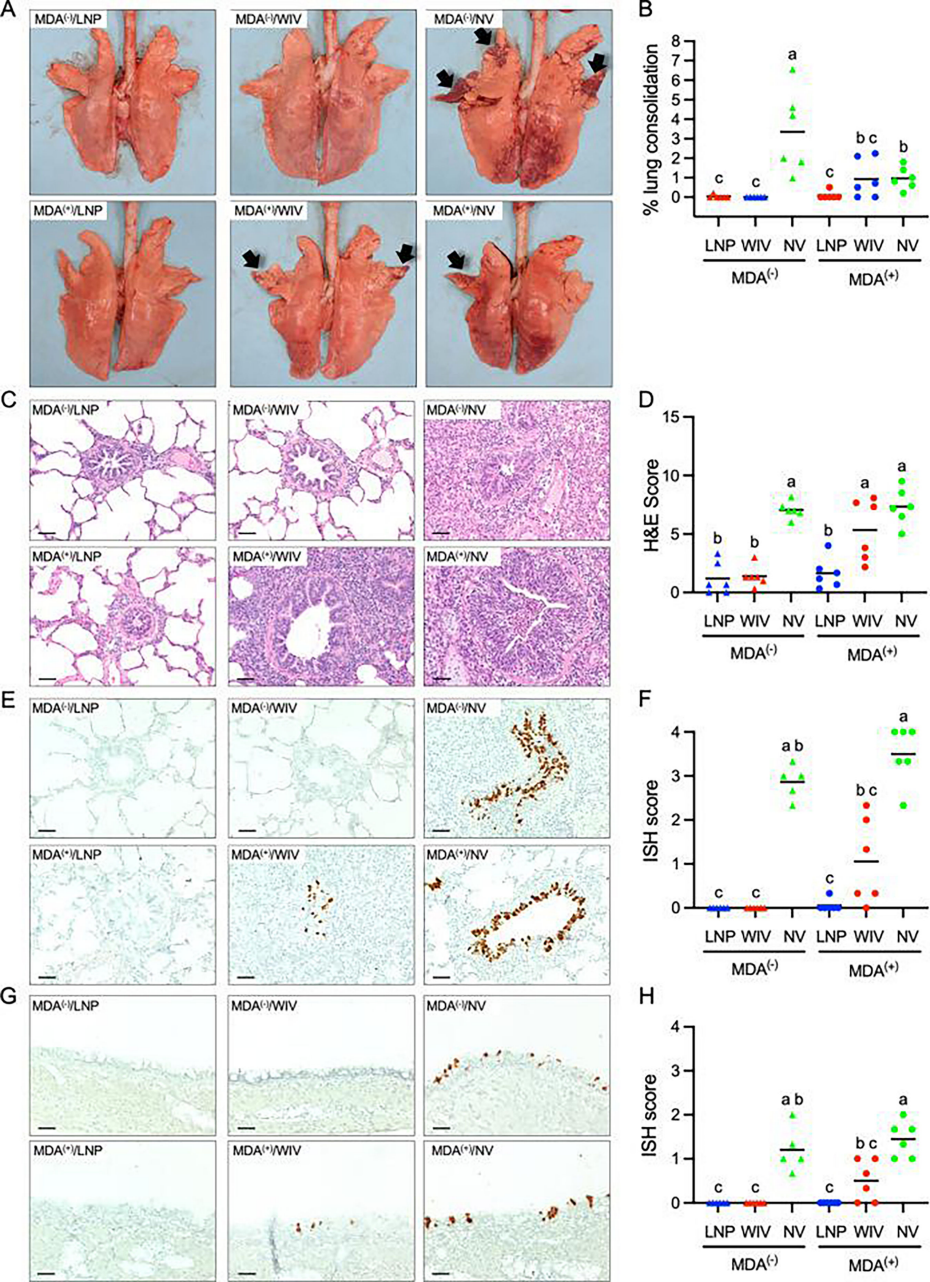
Lung pathology. (**A**) Representative photos of the lungs taken at necropsy. Areas with typical lung consolidation typical of swine IAV infection are indicated by the arrows. (**B**) Percentage of lung surface exhibiting consolidation. (**C**) Representative photos of lung sections stained with H&E. (**D**) Composite score of microscopic lung lesions, ranging from 0 to 15, where 0 indicates normal tissue and 15 represents the most severe lesions. The data represent the mean composite scores from the apical, cranial, and caudal lobes. (**E**) Representative photos of the cranial lung section stained with *in situ* hybridization (ISH) to detect virus-infected cells. (**F**) ISH score of the cranial lobe, based on the abundance of virus-infected cells within a microscopic field at 40× objective lens. (**G**) Representative photos of the tracheal section stained with ISH to detect virus-infected cells. (**H**) ISH score of the tracheal sections based on the abundance of virus-infected cells within a microscopic field at 40× objective lens. (**B, D, F, and H**) Symbols represent data from individual pigs and lines represent group means. Groups with different superscripts indicate statistically significant differences at *P* < 0.05. All microscopic photos were taken using a 20× objective lens. Scale bar = 50 µm.

On day 5 post-challenge with the H1N2 virus, purple-red consolidation typical of swine IAV infection was prominently observed in the lung of pigs in the MDA^(−)^/NV group, particularly in the apical and cardiac lobes, with an average of 3.4% of the total lung surface affected (Fig. 6A and B). In contrast, lung consolidation was not observed in either MDA^(−)^/WIV or MDA^(−)^/LNP groups. The MDA^(+)^/NV group exhibited less severe lung consolidation than the MDA^(−)^/NV group (1% vs 3.4%), suggesting that the presence of MDAs at the time of challenge infection may help mitigate macroscopic lung lesions. Mild consolidation was observed in four of six pigs in the MDA^(+)^/WIV group and in one of six pigs in the MDA^(+)^/LNP group (Fig. 6B).

Despite differences in macroscopic lesions, lung sections from both NV control groups exhibited a similar degree of microscopic lesions, characterized by moderate peribronchiolar lymphocytic cuffing, interstitial pneumonia, epithelial necrosis, and immune cell infiltration into the alveolar septa, along with areas of suppurative bronchiolitis (Fig. 6C). Minimal microscopic alterations were observed in MDA-negative pigs vaccinated with either the LNP-DNA or WIV vaccine, consistent with the absence of macroscopic lesions. In contrast, severe microscopic changes were observed in the lung sections of pigs in the MDA^(+)^/WIV group, with microscopic scores not significantly different from those of the NV control groups (Fig. 6D). Interestingly, only minimal microscopic changes were detected in the MDA-positive pigs vaccinated with the LNP-DNA vaccine. Particularly, the microscopic score for the MDA^(+)^/LNP group was not significantly different from those of the MDA^(−)^/LNP or MDA^(−)^/WIV groups but was significantly lower than the scores of the NV control groups (Fig. 6D).

The ISH assay was performed to detect virus-infected cells in the lung and tracheal tissues. Numerous ISH-positive cells were observed in the tracheal and bronchiolar epithelium of both NV control groups (Fig. 6E and G). In MDA-negative pigs, ISH-positive cells were virtually absent in both lung and tracheal sections, regardless of vaccination with either the LNP or WIV vaccines. Similarly, no ISH-positive cells were found in the lung and tracheal tissues of MDA^(+)^/LNP pigs (Fig. 6F and H). However, ISH-positive cells were clearly detected in the lung and tracheal tissues of MDA^(+)^/WIV pigs, although the number of ISH-positive cells in these tissues was significantly lower than in the NV control groups.

Overall, in the presence of MDA, LNP formulations appear to provide better protection than WIV in terms of reducing lung lesions and virus-infected cells in the lung and tracheal tissues.

## DISCUSSION

Swine IAV is endemic in the United States and many other countries (24). Developing an effective vaccine against IAV in swine faces two major challenges. First, the substantial genetic and antigenic diversity among IAV isolates circulating in swine herds, along with frequent spillover of human- and avian-origin IAV, makes it difficult to develop a universal vaccine that provides broad protection (51). Second, the use of WIV vaccines has resulted in a large proportion of gilts and sows seropositive for IAV, and as a result, their offspring have IAV-specific MDAs (52). These MDAs significantly interfere with the piglets' immune responses to WIV vaccination (12, 38). Therefore, there is a critical need for a new generation of vaccines that can be rapidly updated to match the antigenicity of newly emerging IAV strains in swine herds. Additionally, this new vaccine should be capable of inducing an active immune response in piglets with MDAs. We recently developed an LNP-DNA vaccine against swine IAV that induces protective immunity in MDA-negative pigs with one single dose of immunization (42, 43). In this study, we compared the immunogenicity and protective efficacy of the LNP-DNA vaccine with that of the WIV vaccine in piglets with and without MDAs. Consistent with previous studies (40), we observed that pigs did not exhibit obvious clinical signs, including a febrile response after the challenge with the IAV strain. Therefore, we relied on immunological responses after immunization and viral loads and lung pathology after challenge infection as the key parameters to evaluate vaccine effectiveness.

The HI titers in MDA-positive pigs vaccinated with either the LNP-DNA or WIV vaccine were significantly lower than those observed in their MDA-negative piglets. However, HI titers in the MDA^(+)^/LNP group began to rise from day 21 post-vaccination, whereas the HI titers in the MDA^(+)^/WIV group continued to decline, following a similar pattern observed in the MDA^(+)^/NV group. These findings suggest that the LNP-DNA vaccine is less susceptible to MDA-mediated interference than the WIV vaccine. This observation is noteworthy because the MDA^(+)^/LNP group received only a single vaccine dose while the MDA^(+)^/WIV group received two doses, with the booster dose administered after MDA titers had substantially declined.

The differing effects of MDAs on the HI antibody titers elicited by these two vaccine types may be attributed to the nature of vaccine immunogens. In WIV vaccines, the exogenous viral immunogens are likely to be bound by maternally derived IgG, leading to the formation of antigen-antibody complexes that are recognized by both B cell receptor and inhibitory FcγRIIB receptor on B cells (16). The engagement of the immune complex with the FcγRIIB receptor triggers inhibitory signaling pathways that dampen B cell activation, proliferation, and subsequent antibody production (53). In contrast, the LNP-DNA vaccine delivers DNA encoding the vaccine immunogen into host cells, where it is transcribed and translated to produce antigen intracellularly. We speculate that this endogenous expression limits the formation of immune complexes with MDAs, thereby reducing FcγRIIB-mediated inhibition of B cell responses (54).

The presence of MDA partially interferes with antibody responses in pigs immunized with the LAV vaccine against IAV (36, 37). Specifically, MDA-positive pigs vaccinated with the LAV vaccine exhibited significantly lower titers of serum HI antibodies and mucosal IgG and/or IgA compared to MDA-negative pigs (37). Similarly, in mouse models, active antibody responses to an mRNA-LNP vaccine encoding the HA gene of IAV were also partially hindered by the presence of MDA at the time of vaccination (55). Using a luciferase reporter gene, it has been demonstrated that luciferase expression in MDA-positive pups immunized with the mRNA-LNP vaccine was significantly lower than in MDA-negative controls (56). This finding suggested that the presence of MDA accelerates the clearance of the vaccine immunogen following mRNA-LNP vaccination. We speculate that the partial suppression of HI antibody responses in MDA-positive pigs may result from reduced vaccine antigen expression, as observed in mice vaccinated with the mRNA-LNP vaccine (56), although other mechanisms of MDA interference may also be involved.

Another notable observation in this study is that the LNP-DNA vaccine elicited cellular immunity in both MDA-positive and MDA-negative pigs, whereas the WIV vaccine did not elicit significant cellular immune responses, even in MDA-negative pigs. The frequencies of IFN-γ-SC in PBMCs of pigs vaccinated with WIV vary considerably across studies in the literature (37, 57, 58). Some studies have reported very low IFN-γ-SC numbers (58), while others have observed similar or even higher numbers of IFN-γ-SC in pigs vaccinated with a WIV vaccine than those in pigs vaccinated with a LAV vaccine (37, 57). Notably, all these studies used UV-irradiation for virus inactivation and the same oil-in-water adjuvant, thus ruling out the impact of the inactivation method or adjuvant formulation on the T cell responses. In the present study, the virus was inactivated using BPL and the WIV vaccine was formulated with the same oil-in-water adjuvant. Therefore, the weak peripheral IFN-γ responses observed in MDA-negative pigs vaccinated with the WIV in this study were not unexpected. We speculate that the higher T-cell responses observed in pigs immunized with the LNP-DNA vaccine may be attributed to the efficient delivery of DNA payloads into host cells via the LNP platform, leading to intracellular antigen expression (20), which is more effective at stimulating robust T-cell responses compared to exogenous antigen presentation (59).

In MDA-positive pigs, vaccination with the WIV vaccine failed to prevent virus infection, shedding, or lung microscopic lesions, consistent with findings from previous studies (12, 38). In contrast, MDA-positive pigs vaccinated with the LNP-DNA vaccine exhibited low titers of infectious virus in nasal swabs and undetectable virus in tracheal swabs or BALF. Moreover, the MDA^(+)^/LNP group displayed minimal macroscopic and microscopic lung lesions, similar to MDA-negative pigs that received either the LNP-DNA or WIV vaccine. These findings indicate that the LNP-DNA vaccine induces immunity that prevents viral spread to the lower respiratory tract and lung damage, whereas the WIV vaccine failed to provide any protective effects.

The immune correlates of protection against IAV are complex. HI antibodies are widely considered a reliable correlate of protection (60, 61). In young children vaccinated with either an LAV or an inactivated influenza vaccine, each log_2_ unit increase in HI titers post-vaccination corresponds to approximately a 30% reduction in the risk of infection with the subtype-matched influenza strain (62). In the present study, MDA-negative pigs vaccinated with the WIV vaccine primarily generated an HI antibody response and were fully protected against the homologous strain, further supporting the critical role of HI antibodies in mediating protection. However, the protective threshold of HI titers may vary based on factors such as the animal model, antigenic relatedness between the challenge and vaccine viruses, and the dose and route of infection. Here, the geometric mean HI titer in the MDA^(+)^/WIV group was 1:56 at the time of challenge infection, yet the pigs were not protected against viral replication, shedding, or microscopic lung lesions. Previous studies have shown that HI titers alone do not always predict protection (36, 40). Pigs vaccinated with an LAV were protected against a heterologous challenge infection despite not exhibiting detectable HI antibody levels (36). In contrast, pigs vaccinated with a WIV vaccine had detectable HI antibody levels against the heterologous virus but were not protected after challenge infection (40). Thus, T-cell immunity contributes significantly to vaccine-mediated protection, particularly in heterologous infections (63). In this context, the LNP-DNA vaccine presented in this study, which elicited both HI antibody and T-cell response, especially in pigs with MDAs, may offer a promising platform for developing a vaccine against swine IAV in pigs.

Although the results are encouraging, several aspects remain unaddressed. First, the tissue distribution and persistence of HA antigen expression following LNP-DNA vaccination were not evaluated. A commonly used strategy to study antigen expression in animals is to use LNP-DNA constructs encoding a reporter gene such as luciferase, which allows noninvasive tracking of antigen expression over time (22). However, this approach is unsuitable for large animals like pigs since their body size exceeds the capacity of standard imaging systems. Second, the challenge infection was performed on day 36 post-vaccination. Thus, the long-term durability of the vaccine-induced protection was not evaluated.

In summary, this study demonstrates that the LNP-DNA vaccine induces a balanced antibody and T-cell response in MDA-positive pigs and partially protects the vaccinated pigs against challenge infection with the homologous IAV strain. Since the LNP-DNA vaccine contains only the HA antigen, vaccinated pigs do not develop antibodies against the viral NP. As a result, they can be serologically distinguished from those naturally infected with IAV through antibody tests targeting the viral NP, which is commonly used for IAV serodiagnosis. Overall, these findings highlight the potential of the LNP-DNA vaccine as a tool for improving IAV control in swine populations, particularly in regions where IAV is endemic and MDAs are prevalent.

## Data Availability

All data directly supporting the findings of this study are presented within the article and its supplemental material. The sequences of the H1N2 virus used in this study are available from the National Center for Biotechnology Information, accession number KF715130.1.

## SUPPLEMENTAL MATERIAL

Figure S1 jvi.01123-25-s0001.tiff Representative ELISpot images.

Supplemental legend jvi.01123-25-s0002.docx Legend for Fig. S1.

Tables S1 and S2 jvi.01123-25-s0003.docx HI titers of piglets after vaccination and viral loads in nasal swab after challenge infection.

ASM does not own the copyrights to Supplemental Material that may be linked to, or accessed through, an article. The authors have granted ASM a non-exclusive, world-wide license to publish the Supplemental Material files. Please contact the corresponding author directly for reuse.

## References

[1] Basha S, Surendran N, Pichichero M. 2014. Immune responses in neonates. Expert Rev Clin Immunol 10:1171–1184. doi:10.1586/1744666X.2014.942288 25088080 PMC4407563

[2] Cinicola B, Conti MG, Terrin G, Sgrulletti M, Elfeky R, Carsetti R, Fernandez Salinas A, Piano Mortari E, Brindisi G, De Curtis M, Zicari AM, Moschese V, Duse M. 2021. The protective role of maternal immunization in early life. Front Pediatr 9:638871. doi:10.3389/fped.2021.638871 33996688 PMC8113393

[3] Abdelsattar MM, Rashwan AK, Younes HA, Abdel-Hamid M, Romeih E, Mehanni A-H, Vargas-Bello-Pérez E, Chen W, Zhang N. 2022. An updated and comprehensive review on the composition and preservation strategies of bovine colostrum and its contributions to animal health. Animal Feed Science and Technology 291:115379. doi:10.1016/j.anifeedsci.2022.115379

[4] Langel SN, Paim FC, Lager KM, Vlasova AN, Saif LJ. 2016. Lactogenic immunity and vaccines for porcine epidemic diarrhea virus (PEDV): historical and current concepts. Virus Res 226:93–107. doi:10.1016/j.virusres.2016.05.016 27212686 PMC7111331

[5] Orije MRP, Maertens K, Corbière V, Wanlapakorn N, Van Damme P, Leuridan E, Mascart F. 2020. The effect of maternal antibodies on the cellular immune response after infant vaccination: a review. Vaccine (Auckl) 38:20–28. doi:10.1016/j.vaccine.2019.10.025 31672332

[6] Siegrist C-A, Córdova M, Brandt C, Barrios C, Berney M, Tougne C, Kovarik J, Lambert P-H. 1998. Determinants of infant responses to vaccines in presence of maternal antibodies. Vaccine (Auckl) 16:1409–1414. doi:10.1016/s0264-410x(98)00100-5 9711780

[7] Albrecht P, Ennis FA, Saltzman EJ, Krugman S. 1977. Persistence of maternal antibody in infants beyond 12 months: mechanism of measles vaccine failure. J Pediatr 91:715–718. doi:10.1016/s0022-3476(77)81021-4 909009

[8] Pomorska-Mól M, Markowska-Daniel I, Pejsak Z. 2010. Evaluation of humoral and antigen-specific T-cell responses after vaccination of pigs against pseudorabies in the presence of maternal antibodies. Vet Microbiol 144:450–454. doi:10.1016/j.vetmic.2010.01.015 20153939

[9] Weidinger G, Ohlmann M, Schlereth B, Sutter G, Niewiesk S. 2001. Vaccination with recombinant modified vaccinia virus Ankara protects against measles virus infection in the mouse and cotton rat model. Vaccine (Auckl) 19:2764–2768. doi:10.1016/s0264-410x(00)00531-4 11282186

[10] Patil PK, Sajjanar CM, Natarajan C, Bayry J. 2014. Neutralizing antibody responses to foot-and-mouth disease quadrivalent (type O, A, C and Asia 1) vaccines in growing calves with pre-existing maternal antibodies. Vet Microbiol 169:233–235. doi:10.1016/j.vetmic.2014.01.005 24508311

[11] Klinkenberg D, Moormann RJM, de Smit AJ, Bouma A, de Jong MCM. 2002. Influence of maternal antibodies on efficacy of a subunit vaccine: transmission of classical swine fever virus between pigs vaccinated at 2 weeks of age. Vaccine (Auckl) 20:3005–3013. doi:10.1016/s0042-207x(02)00283-x 12126914

[12] Markowska-Daniel I, Pomorska-Mól M, Pejsak Z. 2011. The influence of age and maternal antibodies on the postvaccinal response against swine influenza viruses in pigs. Vet Immunol Immunopathol 142:81–86. doi:10.1016/j.vetimm.2011.03.019 21501880

[13] Gans HA, Yasukawa LL, Alderson A, Rinki M, DeHovitz R, Beeler J, Audet S, Maldonado Y, Arvin AM. 2004. Humoral and cell-mediated immune responses to an early 2-dose measles vaccination regimen in the United States. J Infect Dis 190:83–90. doi:10.1086/421032 15195246

[14] Siegrist CA, Barrios C, Martinez X, Brandt C, Berney M, Córdova M, Kovarik J, Lambert PH. 1998. Influence of maternal antibodies on vaccine responses: inhibition of antibody but not T cell responses allows successful early prime-boost strategies in mice. Eur J Immunol 28:4138–4148. doi:10.1002/(SICI)1521-4141(199812)28:12<4138::AID-IMMU4138>3.0.CO;2-L 9862350

[15] Niewiesk S. 2014. Maternal antibodies: clinical significance, mechanism of interference with immune responses, and possible vaccination strategies. Front Immunol 5:446. doi:10.3389/fimmu.2014.00446 25278941 PMC4165321

[16] Kim D, Huey D, Oglesbee M, Niewiesk S. 2011. Insights into the regulatory mechanism controlling the inhibition of vaccine-induced seroconversion by maternal antibodies. Blood 117:6143–6151. doi:10.1182/blood-2010-11-320317 21357766 PMC3122939

[17] Vono M, Eberhardt CS, Auderset F, Mastelic-Gavillet B, Lemeille S, Christensen D, Andersen P, Lambert P-H, Siegrist C-A. 2019. Maternal antibodies inhibit neonatal and infant responses to vaccination by shaping the early-life B cell repertoire within germinal centers. Cell Rep 28:1773–1784. doi:10.1016/j.celrep.2019.07.047 31412246

[18] Manickan E, Yu Z, Rouse BT. 1997. DNA immunization of neonates induces immunity despite the presence of maternal antibody. J Clin Invest 100:2371–2375. doi:10.1172/JCI119777 9410917 PMC508435

[19] Hassett DE, Zhang J, Whitton JL. 1997. Neonatal DNA immunization with a plasmid encoding an internal viral protein is effective in the presence of maternal antibodies and protects against subsequent viral challenge. J Virol 71:7881–7888. doi:10.1128/JVI.71.10.7881-7888.1997 9311877 PMC192144

[20] Cui L, Renzi S, Quagliarini E, Digiacomo L, Amenitsch H, Masuelli L, Bei R, Ferri G, Cardarelli F, Wang J, Amici A, Pozzi D, Marchini C, Caracciolo G. 2022. Efficient delivery of DNA using lipid nanoparticles. Pharmaceutics 14:1698. doi:10.3390/pharmaceutics14081698 36015328 PMC9416266

[21] Liao H-C, Shen K-Y, Yang C-H, Chiu F-F, Chiang C-Y, Chai KM, Huang W-C, Ho H-M, Chen Y-H, Huang M-S, Liao C-L, Chen H-W, Huang M-H, Liu S-J. 2024. Lipid nanoparticle-encapsulated DNA vaccine robustly induce superior immune responses to the mRNA vaccine in Syrian hamsters. Molecular Therapy - Methods & Clinical Development 32:101169. doi:10.1016/j.omtm.2023.101169 38187094 PMC10767207

[22] Zhang W, Pfeifle A, Lansdell C, Frahm G, Cecillon J, Tamming L, Gravel C, Gao J, Thulasi Raman SN, Wang L, Sauve S, Rosu-Myles M, Li X, Johnston MJW. 2023. The expression kinetics and immunogenicity of lipid nanoparticles delivering plasmid DNA and mRNA in mice. Vaccines (Basel) 11:1580. doi:10.3390/vaccines11101580 37896985 PMC10610642

[23] Ho T-Y, Hsiang C-Y, Hsiang C-H, Chang T-J. 1998. DNA vaccination induces a long-term antibody response and protective immunity against pseudorabies virus in mice. Arch Virol 143:115–125. doi:10.1007/s007050050272 9505970

[24] Ma W. 2020. Swine influenza virus: current status and challenge. Virus Res 288:198118. doi:10.1016/j.virusres.2020.198118 32798539 PMC7587018

[25] Walia RR, Anderson TK, Vincent AL. 2019. Regional patterns of genetic diversity in swine influenza A viruses in the United States from 2010 to 2016. Influenza Other Respir Viruses 13:262–273. doi:10.1111/irv.12559 29624873 PMC6468071

[26] Anderson TK, Nelson MI, Kitikoon P, Swenson SL, Korslund JA, Vincent AL. 2013. Population dynamics of cocirculating swine influenza A viruses in the United States from 2009 to 2012. Influenza Other Respir Viruses 7 Suppl 4:42–51. doi:10.1111/irv.12193 PMC5655888 24224819

[27] Lewis NS, Russell CA, Langat P, Anderson TK, Berger K. 2016. The global antigenic diversity of swine influenza A viruses. eLife:e12217. doi:10.7554/eLife.12217 27113719 PMC4846380

[28] Ma W, Lager KM, Vincent AL, Janke BH, Gramer MR, Richt JA. 2009. The role of swine in the generation of novel influenza viruses. Zoonoses Public Health 56:326–337. doi:10.1111/j.1863-2378.2008.01217.x 19486316

[29] Powell JD, Abente EJ, Chang J, Souza CK, Rajao DS, Anderson TK, Zeller MA, Gauger PC, Lewis NS, Vincent AL. 2021. Characterization of contemporary 2010.1 H3N2 swine influenza A viruses circulating in United States pigs. Virology (Auckl) 553:94–101. doi:10.1016/j.virol.2020.11.006 33253936

[30] Abente EJ, Anderson TK, Rajao DS, Swenson S, Gauger PC, Vincent AL. 2016. The avian-origin H3N2 canine influenza virus that recently emerged in the United States has limited replication in swine. Influenza Other Respir Viruses 10:429–432. doi:10.1111/irv.12395 27110913 PMC4947940

[31] Rajão DS, Gauger PC, Anderson TK, Lewis NS, Abente EJ, Killian ML, Perez DR, Sutton TC, Zhang J, Vincent AL. 2015. Novel reassortant human-like H3N2 and H3N1 influenza A viruses detected in pigs are virulent and antigenically distinct from swine viruses endemic to the United States. J Virol 89:11213–11222. doi:10.1128/JVI.01675-15 26311895 PMC4645639

[32] Nelson MI, Wentworth DE, Culhane MR, Vincent AL, Viboud C, LaPointe MP, Lin X, Holmes EC, Detmer SE. 2014. Introductions and evolution of human-origin seasonal influenza a viruses in multinational swine populations. J Virol 88:10110–10119. doi:10.1128/JVI.01080-14 24965467 PMC4136342

[33] Nelson MI, Stratton J, Killian ML, Janas-Martindale A, Vincent AL. 2015. Continual reintroduction of human pandemic H1N1 influenza A viruses into swine in the United States, 2009 to 2014. J Virol 89:6218–6226. doi:10.1128/JVI.00459-15 25833052 PMC4474294

[34] Sandbulte MR, Spickler AR, Zaabel PK, Roth JA. 2015. Optimal use of vaccines for control of influenza A virus in swine. Vaccines (Basel) 3:22–73. doi:10.3390/vaccines3010022 26344946 PMC4494241

[35] Vincent AL, Lager KM, Janke BH, Gramer MR, Richt JA. 2008. Failure of protection and enhanced pneumonia with a US H1N2 swine influenza virus in pigs vaccinated with an inactivated classical swine H1N1 vaccine. Vet Microbiol 126:310–323. doi:10.1016/j.vetmic.2007.07.011 17719188

[36] Vincent AL, Ma W, Lager KM, Richt JA, Janke BH, Sandbulte MR, Gauger PC, Loving CL, Webby RJ, García-Sastre A. 2012. Live attenuated influenza vaccine provides superior protection from heterologous infection in pigs with maternal antibodies without inducing vaccine-associated enhanced respiratory disease. J Virol 86:10597–10605. doi:10.1128/JVI.01439-12 22811541 PMC3457301

[37] Sandbulte MR, Platt R, Roth JA, Henningson JN, Gibson KA, Rajão DS, Loving CL, Vincent AL. 2014. Divergent immune responses and disease outcomes in piglets immunized with inactivated and attenuated H3N2 swine influenza vaccines in the presence of maternally-derived antibodies. Virology (Auckl) 464–465:45–54. doi:10.1016/j.virol.2014.06.027 25043588

[38] Kitikoon P, Nilubol D, Erickson BJ, Janke BH, Hoover TC, Sornsen SA, Thacker EL. 2006. The immune response and maternal antibody interference to a heterologous H1N1 swine influenza virus infection following vaccination. Vet Immunol Immunopathol 112:117–128. doi:10.1016/j.vetimm.2006.02.008 16621020

[39] Abente EJ, Rajao DS, Santos J, Kaplan BS, Nicholson TL, Brockmeier SL, Gauger PC, Perez DR, Vincent AL. 2018. Comparison of adjuvanted-whole inactivated virus and live-attenuated virus vaccines against challenge with contemporary, antigenically distinct H3N2 influenza A viruses. J Virol 92:e01323-18. doi:10.1128/JVI.01323-18 PMC6206469 30185589

[40] Loving CL, Lager KM, Vincent AL, Brockmeier SL, Gauger PC, Anderson TK, Kitikoon P, Perez DR, Kehrli ME. 2013. Efficacy in pigs of inactivated and live attenuated influenza virus vaccines against infection and transmission of an emerging H3N2 similar to the 2011-2012 H3N2v. J Virol 87:9895–9903. doi:10.1128/JVI.01038-13 23824815 PMC3754103

[41] Sharma A, Zeller MA, Li G, Harmon KM, Zhang J, Hoang H, Anderson TK, Vincent AL, Gauger PC. 2020. Detection of live attenuated influenza vaccine virus and evidence of reassortment in the U.S. swine population. J Vet Diagn Invest 32:301–311. doi:10.1177/1040638720907918 32100644 PMC7081507

[42] Nguyen TN, Lai DC, Sillman S, Petro-Turnquist E, Weaver EA, Vu HLX. 2024. Lipid nanoparticle-encapsulated DNA vaccine confers protection against swine and human-origin H1N1 influenza viruses. mSphere 9:e0028324. doi:10.1128/msphere.00283-24 39087764 PMC11351038

[43] Nguyen TN, Kumari S, Sillman S, Chaudhari J, Lai DC, Vu HLX. 2023. A single-dose intramuscular immunization of pigs with lipid nanoparticle DNA vaccines based on the hemagglutinin antigen confers complete protection against challenge infection with the homologous influenza virus strain. Vaccines (Basel) 11:1596. doi:10.3390/vaccines11101596 37896997 PMC10611089

[44] Roces CB, Lou G, Jain N, Abraham S, Thomas A, Halbert GW, Perrie Y. 2020. Manufacturing considerations for the development of lipid nanoparticles using microfluidics. Pharmaceutics 12:1095. doi:10.3390/pharmaceutics12111095 33203082 PMC7697682

[45] Gauger PC, Loving CL, Khurana S, Lorusso A, Perez DR, Kehrli ME Jr, Roth JA, Golding H, Vincent AL. 2014. Live attenuated influenza A virus vaccine protects against A(H1N1)pdm09 heterologous challenge without vaccine associated enhanced respiratory disease. Virology (Auckl) 471–473:93–104. doi:10.1016/j.virol.2014.10.003 25461535

[46] Halbur PG, Paul PS, Frey ML, Landgraf J, Eernisse K, Meng XJ, Lum MA, Andrews JJ, Rathje JA. 1995. Comparison of the pathogenicity of two US porcine reproductive and respiratory syndrome virus isolates with that of the Lelystad virus. Vet Pathol 32:648–660. doi:10.1177/030098589503200606 8592800

[47] Gauger PC, Vincent AL, Loving CL, Henningson JN, Lager KM, Janke BH, Kehrli ME, Roth JA. 2012. Kinetics of lung lesion development and pro-inflammatory cytokine response in pigs with vaccine-associated enhanced respiratory disease induced by challenge with pandemic (2009) A/H1N1 influenza virus. Vet Pathol 49:900–912. doi:10.1177/0300985812439724 22461226

[48] Sun H, Sur J-H, Sillman S, Steffen D, Vu HLX. 2019. Design and characterization of a consensus hemagglutinin vaccine immunogen against H3 influenza A viruses of swine. Vet Microbiol 239:108451. doi:10.1016/j.vetmic.2019.108451 31767095

[49] Oh T, Do DT, Lai DC, Nguyen TC, Vo HV, Chae C. 2021. Age-related viral load and severity of systemic pathological lesions in acute naturally occurring African swine fever virus genotype II infections. Comp Immunol Microbiol Infect Dis 79:101709. doi:10.1016/j.cimid.2021.101709 34543808

[50] Gauger PC, Vincent AL. 2020. Serum virus neutralization assay for detection and quantitation of serum neutralizing antibodies to influenza A virus in swine, p 321–333. Methods and Protocols. Springer, Animal Influenza Virus. 10.1007/978-1-0716-0346-8_23 32170698

[51] Vincent AL, Perez DR, Rajao D, Anderson TK, Abente EJ, Walia RR, Lewis NS. 2017. Influenza A virus vaccines for swine. Vet Microbiol 206:35–44. doi:10.1016/j.vetmic.2016.11.026 27923501 PMC8609643

[52] Rajao DS, Anderson TK, Gauger PC, Vincent AL. 2014. Pathogenesis and vaccination of influenza A virus in swine. Curr Top Microbiol Immunol 385:307–326. doi:10.1007/82_2014_391 25033752

[53] Nimmerjahn F, Ravetch JV. 2008. Fcgamma receptors as regulators of immune responses. Nat Rev Immunol 8:34–47. doi:10.1038/nri2206 18064051

[54] Kozak M, Hu J. 2024. DNA vaccines: their formulations, engineering and delivery. Vaccines (Basel) 12:71. doi:10.3390/vaccines12010071 38250884 PMC10820593

[55] Willis E, Pardi N, Parkhouse K, Mui BL, Tam YK, Weissman D, Hensley SE. 2020. Nucleoside-modified mRNA vaccination partially overcomes maternal antibody inhibition of de novo immune responses in mice. Sci Transl Med 12:eaav5701. doi:10.1126/scitranslmed.aav5701 31915303 PMC7339908

[56] Dangi T, Sanchez S, Awakoaiye B, Lew MH, Irani N, Penaloza-MacMaster P. 2024. Breast milk-derived antibodies impair vaccine immunity in suckling mice. J Immunol 213:612–618. doi:10.4049/jimmunol.2400277 39007643 PMC11333162

[57] Olson ZF, Sandbulte MR, Souza CK, Perez DR, Vincent AL, Loving CL. 2017. Factors affecting induction of peripheral IFN-γ recall response to influenza A virus vaccination in pigs. Vet Immunol Immunopathol 185:57–65. doi:10.1016/j.vetimm.2017.01.009 28242003

[58] Loving CL, Vincent AL, Pena L, Perez DR. 2012. Heightened adaptive immune responses following vaccination with a temperature-sensitive, live-attenuated influenza virus compared to adjuvanted, whole-inactivated virus in pigs. Vaccine (Auckl) 30:5830–5838. doi:10.1016/j.vaccine.2012.07.033 PMC3743435 22835742

[59] Laczkó D, Hogan MJ, Toulmin SA, Hicks P, Lederer K, Gaudette BT, Castaño D, Amanat F, Muramatsu H, Oguin TH 3rd, et al.. 2020. A single immunization with nucleoside-modified mRNA vaccines elicits strong cellular and humoral immune responses against SARS-CoV-2 in mice. Immunity 53:724–732. doi:10.1016/j.immuni.2020.07.019 32783919 PMC7392193

[60] Memoli MJ, Shaw PA, Han A, Czajkowski L, Reed S, Athota R, Bristol T, Fargis S, Risos K, Powers JH, Davey RT Jr, Taubenberger JK. 2016. Evaluation of antihemagglutinin and antineuraminidase antibodies as correlates of protection in an influenza A/H1N1 virus healthy human challenge model. mBio 7:e00417–16. doi:10.1128/mBio.00417-16 27094330 PMC4959521

[61] Black S, Nicolay U, Vesikari T, Knuf M, Del Giudice G, Della Cioppa G, Tsai T, Clemens R, Rappuoli R. 2011. Hemagglutination inhibition antibody titers as a correlate of protection for inactivated influenza vaccines in children. Pediatr Infect Dis J 30:1081–1085. doi:10.1097/INF.0b013e3182367662 21983214

[62] Yegorov S, Brewer A, Cyr L, Ward BJ, Pullenayegum E, Miller MS, Loeb M. 2025. Hemagglutination-inhibition antibodies and protection against influenza elicited by inactivated and live attenuated vaccines in children. J Infect Dis 231:e308–e316. doi:10.1093/infdis/jiae489 39504434 PMC11841627

[63] Sridhar S. 2016. Heterosubtypic T-cell immunity to influenza in humans: challenges for universal T-cell influenza vaccines. Front Immunol 7:195. doi:10.3389/fimmu.2016.00195 27242800 PMC4871858

