# Supplementary figures and images for: Lipid nanoparticle-encapsulated DNA vaccine induces balanced antibody and T-cell responses in pigs with maternally derived antibodies
Source: J Virol. 2025 Oct 9;99(11):e01123-25. doi: 10.1128/jvi.01123-25 (PMC12645943; doi:10.1128/jvi.01123-25)

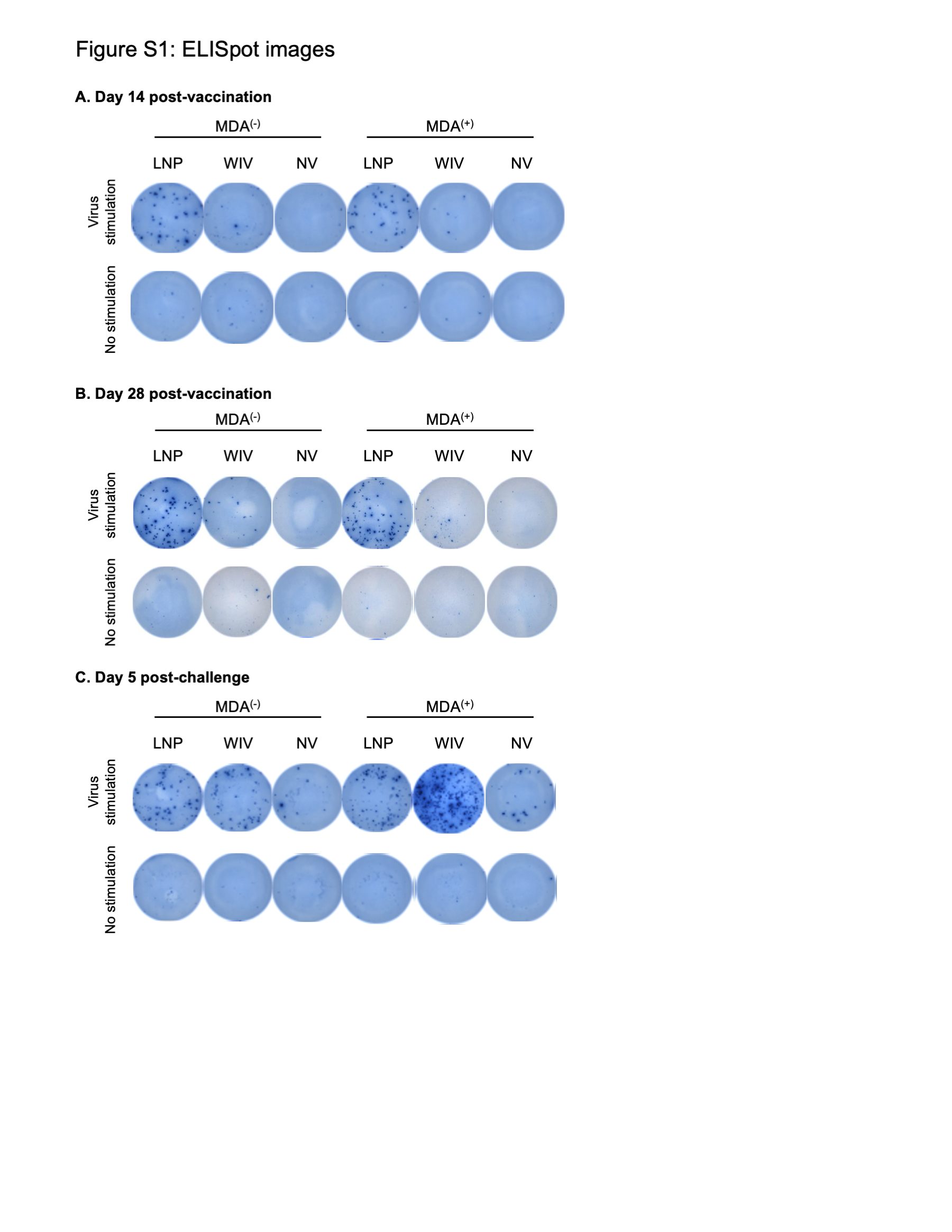

Supplement: Figure S1 — Representative ELISpot images. [file jvi.01123-25-s0001.tiff]
