# Supplementary material for: Lipid nanoparticle-encapsulated DNA vaccine induces balanced antibody and T-cell responses in pigs with maternally derived antibodies
Source: J Virol. 2025 Oct 9;99(11):e01123-25. doi: 10.1128/jvi.01123-25 (PMC12645943; doi:10.1128/jvi.01123-25)
Supplement: Supplemental legend — Legend for Fig. S1. [file jvi.01123-25-s0002.docx]

**Figure S1. Representative images of the IFN-γ ELISpot assay.**
PBMCs were collected at different time points post-vaccination, and the IFN-γ ELISpot assay was performed as described in the Materials and Methods section. Shown are representative images of IFN-γ spots from samples collected on day 14 (A), day 28 (B), and day 41 (C) post-vaccination.
