# Supplementary material for: Lipid nanoparticle-encapsulated DNA vaccine induces balanced antibody and T-cell responses in pigs with maternally derived antibodies
Source: J Virol. 2025 Oct 9;99(11):e01123-25. doi: 10.1128/jvi.01123-25 (PMC12645943; doi:10.1128/jvi.01123-25)
Supplement: Tables S1 and S2 — HI titers of piglets after vaccination and viral loads in nasal swab after challenge infection. [file jvi.01123-25-s0003.docx]

**Table S1: HI titers in piglets after vaccination**

| **Day post-vaccination** | **Treatment groups** | | | | | |
| --- | --- | --- | --- | --- | --- | --- |
|  | **MDA^(-)^/LNP** | **MDA^(-)^/WIV** | **MDA^(-)^/NV** | **MDA^(+)^/LNP** | **MDA^(+)^/WIV** | **MDA^(+)^/NV** |
| 0 | 2.32 ± 0 ^b^ | 2.32 ± 0 ^b^ | 2.32 ± 0 ^b^ | 7.82 ± 0.5 ^a^ | 8.16 ± 0.69 ^a^ | 7.66 ± 0.47 ^a^ |
| 14 | 6.32 ± 0.58 ^a^ | 4.16 ± 0.37 ^b^ | 2.32 ± 0 ^c^ | 6.82 ± 0.5 ^a^ | 6.32 ± 1 ^a^ | 6.99 ± 0.47 ^a^ |
| 21 | 8.16 ± 0.37 ^a^ | 5.66 ± 1.25 ^b^ | 2.32 ± 0 ^c^ | 6.49 ± 0.9 ^b^ | 6.49 ± 0.9 ^b^ | 6.32 ± 0.58 ^b^ |
| 28 | 9.49 ± 0.37 ^a^ | 9.82 ± 0.5 ^a^ | 2.32 ± 0 ^d^ | 6.66 ± 0.75 ^b^ | 6.32 ± 0.82 ^bc^ | 5.49 ± 0.37 ^c^ |
| 35 | 9.82 ± 0.5 ^a^ | 9.16 ± 0.69 ^a^ | 2.32 ± 0 ^d^ | 6.82 ± 1.12 ^b^ | 5.82 ± 0.5 ^c^ | 5.32 ± 0.58 ^c^ |
| 41 | 9.82 ± 0.5 ^a^ | 8.32 ± 1 ^b^ | 2.32 ± 0 ^f^ | 7.16 ± 0.69 ^c^ | 5.66 ± 0.75 ^d^ | 4.49 ± 0.37 ^d^ |

Data are presented as mean log_2_ HI titer ± standard deviation.

Within a row, means with different superscripts are significantly different at p < 0.05.

**Table S2. Viral loads in nasal swabs collected daily during the 5 days post-challenge infection with the H1N2 virus**

| **Day post-challenge** | **Treatment groups** | | | | | |
| --- | --- | --- | --- | --- | --- | --- |
|  | **MDA^(-)^/LNP** | **MDA^(-)^/WIV** | **MDA^(-)^/NV** | **MDA^(+)^/LNP** | **MDA^(+)^/WIV** | **MDA^(+)^/NV** |
| 0 | 0 ± 0 | 0 ± 0 | 0 ± 0 | 0 ± 0 | 0 ± 0 | 0 ± 0 |
| 1 | 0 ± 0 ^c^ | 0.29 ± 0.71 ^bc^ | 2.25 ± 0.57 ^a^ | 0.29 ± 0.71 ^bc^ | 1.92 ± 1.57 ^ab^ | 2 ± 1.7 ^a^ |
| 2 | 0 ± 0 ^b^ | 0 ± 0 ^b^ | 3.37 ± 0.26 ^a^ | 1.37 ± 1.56 ^b^ | 3.62 ± 0.61 ^a^ | 4.29 ± 0.64 ^a^ |
| 3 | 0 ± 0 ^c^ | 0 ± 0 ^c^ | 4 ± 1.31 ^ab^ | 2.63 ± 2.38 ^b^ | 4.29 ± 0.42 ^ab^ | 4.46 ± 1.22 ^a^ |
| 4 | 0 ± 0 ^c^ | 0 ± 0 ^c^ | 3.92 ± 0.85 ^a^ | 2.15 ± 1.79 ^b^ | 5.26 ± 0.46 ^a^ | 4.12 ± 0.85 ^a^ |
| 5 | 0 ± 0 ^b^ | 0 ± 0 ^b^ | 4.46 ± 1.07 ^a^ | 1.62 ± 1.91 ^b^ | 3.92 ± 0.63 ^a^ | 4.96 ± 0.49 ^a^ |

Data are presented as mean log₁₀ TCID₅₀/mL ± standard deviation.

Within a row, means with different superscripts are significantly different at p < 0.05.
